# Converting loss‐on‐ignition to organic carbon content in arable topsoil: pitfalls and proposed procedure

**DOI:** 10.1111/ejss.12558

**Published:** 2018-05-03

**Authors:** J. L. Jensen, B. T. Christensen, P. Schjønning, C. W. Watts, L. J. Munkholm

**Affiliations:** ^1^ Department of Agroecology Aarhus University, AU‐Foulum 8830 Tjele Denmark; ^2^ Department of Sustainable Agriculture Sciences Rothamsted Research West Common, Harpenden Hertfordshire AL5 2JQ UK

## Abstract

Assessments of changes in soil organic carbon (SOC) stocks depend heavily on reliable values of SOC content obtained by automated high‐temperature C analysers. However, historical as well as current research often relies on indirect SOC estimates such as loss‐on‐ignition (LOI). In this study, we revisit the conversion of LOI to SOC using soil from two long‐term agricultural field experiments and one arable field with different contents of SOC, clay and particles <20 μm (Fines20). Clay‐, silt‐ and sand‐sized fractions were isolated from the arable soil. Samples were analysed for texture, LOI (500°C for 4 hours) and SOC by dry combustion. For a topsoil with 2 g C and 30 g clay 100 g^−1^ soil, converting LOI to SOC by the conventional factor 0.58 overestimated the SOC stock by 45 Mg C ha^−1^. The error increased with increasing contents of clay and Fines20. Converting LOI to SOC by a regression model underestimated the SOC stock by 5 Mg C ha^−1^ at small clay and Fines20 contents and overestimated the SOC stock by 8 Mg C ha^−1^ at large contents. This was due to losses of structural water from clay minerals. The best model to convert LOI to SOC incorporated clay content. Evaluating this model against an independent dataset gave a root mean square error and mean error of 0.295 and 0.125 g C 100 g^−1^, respectively. To avoid misleading accounts of SOC stocks in agricultural soils, we recommend re‐analysis of archived soil samples for SOC using high‐temperature dry combustion methods. Where archived samples are not available, accounting for clay content improves conversion of LOI to SOC considerably. The use of the conventional conversion factor 0.58 is antiquated and provides misleading estimates of SOC stocks.

**Highlights:**

- Assessment of SOC contents is often based on less accurate methods such as LOI.
- Reliable accounts of changes in SOC stocks remain high on the agenda (4‰ initiative).
- Conversion of LOI to SOC is considerably improved by accounting for clay content.
- Converting LOI to SOC by the conventional factor 0.58 leads to grossly overestimated SOC stocks.

## Introduction

Accounting for changes in soil organic carbon (SOC) induced by changes in climate, land use and soil management remains high on the agenda, as exemplified by the 4 per mille initiative launched at the recent COP‐21 conference in Paris (Minasny *et al*., 2017). This global research initiative aims at a relative annual increase in SOC of 0.4% in the top 40 cm of soil. Changes in SOC stocks occur slowly and over long periods, therefore verification of changes involves present as well as historical accounts of SOC. Verification of changes in SOC stocks on global scales are not always well described in terms of sources of SOC content data and methods used for determination of SOC (Stockmann *et al*., 2015; Hengl *et al*., 2017). Accurate and precise determination of SOC contents is fundamental for reliable estimates of SOC stocks (Goidts *et al*., 2009; Conant *et al.,* 2011; Schrumpf *et al*., 2011); this can be obtained by automated, high‐temperature dry combustion methods (Chatterjee *et al*., 2009).

Loss‐on‐ignition (LOI), however, remains a widely used method for assessing SOC in agricultural and forest soils, with LOI being converted to SOC either by a fixed conversion factor or by regression analyses (Konen *et al*., 2002; De Vos *et al*., 2005; Salehi *et al*., 2011; Reynolds *et al*., 2013). The basic assumption is that LOI is due only to combustion of soil organic matter (SOM) and that the content of SOC in SOM is constant (Christensen & Malmros, 1982). No standard protocol exists for LOI analysis, but it is well documented that LOI is affected by ignition temperature, duration of ignition and ignited sample mass (Abella & Zimmer, 2007; Salehi *et al*., 2011; Hoogsteen *et al*., 2015). Further, structural water loss (SWL) from soil minerals may contribute significantly to LOI (Sun *et al*., 2009; Hoogsteen *et al*., 2015) and the validity of the conventional LOI‐to‐SOC conversion factor of 0.58, although widely used, remains dubious (Pribyl, 2010). When LOI and SOC are both measured, regression models for converting LOI to SOC have been proposed (Grewal *et al*., 1991; De Vos *et al*., 2005; Abella & Zimmer, 2007). Regression models based on less accurate analytical approaches, such as dichromate oxidation followed by titration, and soils with confounding effects from differences in clay mineralogy have been found to be less reliable (Howard & Howard, 1990).

In our current research attempting to define critical small SOM contents for soil structural properties based on the clay content (< 2 μm)/SOC and particles < 20 μm (Fines20)/SOC ratios (Schjønning *et al*., 2012; Getahun *et al*., 2016; Jensen *et al*., 2017a), it is essential to have access to reliable values of SOC content. The combined fraction of clay plus silt (particles <20 µm) is denoted Fines20. As a ‘spin‐off’ from this research, we revisited the conversion of LOI to SOC. Data for temperate zone arable topsoil with different contents of SOC were collected from long‐term agricultural field experiments with contrasting management at Askov (Denmark) and Rothamsted (UK), and from a texture gradient in a farmer's field at Lerbjerg (Denmark) with uniform management and mineralogy. These fields had large ranges in LOI, SOC, clay and Fines20, making them representative of arable soils with respect to these properties. We also included clay‐, silt‐ and sand‐sized fractions isolated from Lerbjerg soil samples.

## Materials and methods

### Rothamsted Highfield ley–arable experiment

Soil texture and SOC data for the Highfield experiment at Rothamsted Research, UK (51°80′N, 00°36′W), were extracted from Jensen *et al*. (2017b). This experiment is on a silt loam soil belonging to the Batcombe series; the parent material includes a silty (loess‐containing) deposit overlying and mixed with clay‐with‐flints (Avery & Catt, 1995). The soil was classified as an Aquic Paludalf (Soil Survey Staff, 2014) and Chromic Luvisol (IUSS Working Group WRB, 2015). The clay fraction is dominated by smectite, mica and kaolinite, with traces of feldspar, chlorite and crystalline and amorphous ferric oxides (Avery & Catt, 1995). Bulk soil was taken in spring 2015 from the 6–15‐cm layer of four different treatments: bare fallow maintained free of vegetation since 1959, arable rotation with winter cereals (winter wheat (*Triticum aestivum* L.) and winter oat (*Avena sativa* L.)) since 1948, ley–arable rotation with 3‐year grass–clover ley (meadow fescue (*Festuca pratensis* L.), timothy‐grass (*Phleum pratense* L.) and white clover (*Trifolium repens* L.)) followed by 3 years under arable management (as arable rotation) since 1948 and grassland ploughed and reseeded to grass (predominantly rye grass, *Lolium perenne* L.) in 1948. Soil was sampled from three positions within each of four replicate plots, providing 48 samples. Jensen *et al*. (2017b) provide further details.

### Askov long‐term experiment on animal manure and mineral fertilizers (Askov‐LTE)

Data on soil texture and SOC for the Askov‐LTE in southern Denmark (55°28′N, 09°07′E) were retrieved from Jensen *et al*. (2017a). This experiment was established in 1894 on a sandy loam soil. The parent material comprises terminal morain deposits from the Weichselian glaciation stage. The soil was classified as a Ultic Hapludalf (Soil Survey Staff, 2014) and Aric Haplic Luvisol (IUSS Working Group WRB, 2015). The clay fraction is dominated by illite, kaolinite, quartz and smectite, with traces of vermiculite, Al‐Fe‐oxyhydroxides, feldspar and chlorite. Following harvest of winter wheat (*Triticum aestivum* L.), bulk soil was sampled in autumn 2014 from the 6–15‐cm layer of four different treatments in the B5 field: unfertilized, ½ mineral fertilizer (since 1923), 1 mineral fertilizer and 1½ animal manure. Nutrient addition rate 1 corresponds to 150 kg total‐N ha^−1^, 30 kg P ha^−1^ and 120 kg K ha^−1^. Three replicate plots of each treatment were sampled, providing 12 samples. Further details are given in Jensen *et al*. (2017a).

### Lerbjerg textural gradient

Soil was sampled from a naturally occurring textural gradient located in an arable field at Lerbjerg, Denmark (56°22′N, 09°59′E). The Lerbjerg field has a uniform parent material (Weichselian morainic deposits) but varies widely in both texture and SOC content. The clay fraction is dominated by illite, smectite and vermiculite, with traces of kaolinite, quartz and feldspar (Schjønning *et al*., 1999). Bulk soil from the 5*–*10‐cm layer was sampled in autumn 2015 at 16 locations along the texture gradient following harvest of oil‐seed rape (*Brassica napus* L.).

### Lerbjerg soil particle‐size fractions

Archived samples of soil particle‐size fractions from Lerbjerg (Schjønning & de Jonge, 1999) were used to estimate soil mineral structural water loss (SWL) from clay‐ (< 2 μm), silt‐ (2*–*63 μm) and sand‐sized (63*–*2000 μm) soil components. Soil samples were fully dispersed with an ultrasonic probe (300 W for 15 minutes), and the size fractions were isolated by a combination of sedimentation in water and dry sieving. Schjønning & de Jonge (1999) describe the protocol for particle‐size fractionation in detail.

### Determination of clay, silt, loss‐on‐ignition and soil organic carbon

Clay (< 2 μm) and silt (2*–*20 μm) contents of air‐dried soil (< 2 mm) were determined by the hydrometer method for Highfield and Askov, and the pipette method for Lerbjerg, both described by Gee & Or (2002). Samples for determination of clay and silt were treated with hydrogen peroxide to remove SOM. The presence of carbonates was tested by adding a few droplets of 10% HCl, but none was found. Loss‐on‐ignition was determined on the oven‐dried subsamples of bulk soil and soil size fractions. Five grams of air‐dry soil was added to previously ignited and weighed porcelain crucibles, dried at 105°C for 24 hours in a ventilated oven, cooled in a desiccator and weighed again. Residual water content (RWC) was calculated as the difference between the air‐dry and oven‐dry weights and related to oven‐dry soil. Finally, the crucibles were ignited at 500°C for 4 hours in a muffle furnace (Thermolyne Largest Tabletop Muffle Furnace, Thermo Fisher Scientific, Waltham, MA, USA). After ignition, the crucibles were cooled in a desiccator and weighed. The LOI was calculated as the difference between the oven‐dry weight before and after ignition and related to oven‐dry soil. The SOC content was determined by high‐temperature dry combustion using ball‐milled subsamples of air‐dried soil (< 2 mm). A Thermo Flash 2000 NC Soil Analyser (Thermo Fisher Scientific, Waltham MA, USA) was used for soil size fractions from Lerbjerg and bulk soil from Highfield and Askov, and an ELTRA Helios C‐Analyser (ELTRA GmbH, Haan, NRW, Germany) was used for bulk soil from Lerbjerg. Results for SOC, LOI and soil size fractions are expressed as g 100 g^−1^ oven‐dry soil (105°C for 24 hours).

### Evaluation dataset

The regression model developed to convert LOI to SOC was evaluated using a study reporting data on LOI‐450 (450°C for 5 hours), LOI‐550 (550°C for 5 hours), SOC (high‐temperature dry combustion) and clay content (Grewal *et al*., 1991). This study was used because it focused on arable soil, measured SOC by high‐temperature dry combustion, reported clay content, used an LOI protocol close to ours and reported raw data in tabulated form. The study was based on 40 samples from cultivated soils and grasslands in New Zealand, including topsoil and subsoil. The samples were from eight different fields, of which five differed in parent material. Our study was based solely on data from topsoil; therefore, subsoil samples (> 35 cm depth) in the evaluation dataset were not considered, reducing the evaluation dataset to 31 samples. This subset of data had a range of values for SOC, clay, LOI‐450 and LOI‐550 from 0.75 to 6.33, 17 to 57, 2.64 to 15.19 and 3.35 to 15.94 g 100 g^−1^, respectively.

### Statistics

Linear regression was applied using the R‐project software package version 3.1.1 (R Core Team, 2017). The variance inflation factor (VIF) was calculated when more than one predictor was used in the regression. The VIF expresses the degree of multicollinearity among the predictors. The upper threshold value for non‐erroneous conclusions from multiple regressions has been set previously to 5 (Rogerson, 2001) or 10 (Kutner *et al*., 2004). For models with more than one predictor and an intercept term, the adjusted coefficient of determination (*R* ^2^) is reported. The *R* ^2^ was calculated as 1–SS_res_/SS_tot_ for models without intercept, where SS_res_ is from the model without intercept and SS_tot_ from the model with intercept. Akaike's information criterion (AIC) was used to compare models with different numbers of parameters (Akaike, 1973). A smaller or more negative AIC indicates better model performance. The root mean square error (RMSE) and mean error (ME) were calculated to evaluate model performance:

(1) $$\text{RMSE}=\sqrt{\frac{1}{m}\sum_{i=1}^{m}d_{i}^{2}},$$

(2) $$\mathrm{ME}=\frac{1}{m}\sum_{i=1}^{m}d_{i},$$

where *d*_*i*_ is the difference between the predicted and measured SOC content and *m* is the sample size.

## Results

The soils differed in SOC content because of long‐term contrasting management at Highfield and Askov, and soil topography at Lerbjerg (Table 1). Clay and Fines20 contents varied little at Highfield and Askov, whereas samples from Lerbjerg reflected a wide texture gradient.

**Table 1:** Soil organic carbon (SOC), loss‐on‐ignition (LOI), clay (< 2 μm) and mineral particles < 20 μm (Fines20) for bulk soils and particle‐size fractions from Lerbjerg

		SOC	LOI	Clay	Fines20
Sample	*n*	Mean, minimum and maximum / g 100 g^−1^ soil			
Highfield (UK)	48	1.93 (0.78–3.94)	5.84 (3.62–9.80)	25 (22–32)	50 (47–57)
Askov (Denmark)	12	1.09 (0.86–1.37)	2.90 (2.40–3.55)	9 (9–10)	19 (17–20)
Lerbjerg (Denmark)	16	2.37 (1.06–4.14)	7.11 (3.34–12.08)	38 (10–73)	49 (15–91)
Clay fraction < 2 μm	4	1.81 (1.44–2.17)	7.63 (7.12–8.08)	98 (98–99)	NA
Silt fraction 2–63 μm	4	2.00 (1.82–2.27)	4.76 (4.57–4.92)	0	NA
Sand fraction 63–2000 μm	4	0.14 (0.04–0.20)	0.42 (0.22–0.65)	0	0

*n*, number of samples; NA, not applicable.

The VIF, calculated for the combination of LOI and clay, was 1.02, 1.18 and 6.83 for Highfield, Askov and Lerbjerg, respectively. For LOI and Fines20, the corresponding VIF was 1.00, 1.06 and 6.58. Although the use of VIF threshold values has been questioned (O'Brien, 2007), the degree of multicollinearity among predictors in the regressions was small for Highfield and Askov and allowed the use of both in the regression analysis. We recognize that the VIF value for Lerbjerg was on the limit of multicollinearity.

The RWC ranged from 0.9 to 6.2 g 100 g^−1^ oven‐dry soil and increased linearly with increasing contents of SOC and clay content (data not shown). For the soil with the largest content of clay (69 g clay 100 g^−1^) and SOC (4.14 g C 100 g^−1^), neglecting the correction for RWC underestimates SOC by 0.26 g C 100 g^−1^.

**Figure 1:**
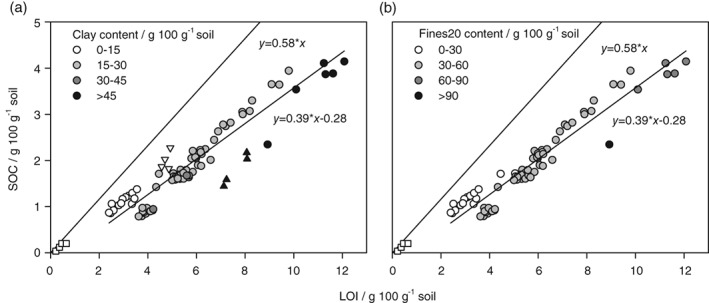
Soil organic carbon (SOC) as a function of loss‐on‐ignition (LOI) for (a) samples grouped by soil clay content (n = 88) and (b) samples grouped by soil mineral particles < 20 μm (Fines20) content (n = 80). The clay‐ (< 2 μm), silt‐ (2–63 μm) and sand‐sized (63–2000 μm) fractions from the Lerbjerg site are shown with triangle up, triangle down and square symbols, respectively. Because of different size limits for silt isolated from Lerbjerg, panel (b) does not include Fines20 from Lerbjerg. The line representing the conventional relation between LOI and SOC (SOC = 0.58 × LOI) is also shown.

There was a strong positive relation between LOI and SOC (SOC = 0.39 × LOI − 0.28; Figure 1). In general, the sandy soils are above the regression line, whereas the clayey soils are below. The clay (< 2 μm), silt (2*–*63 μm) and sand (63*–*2000 μm) fractions were not included in the linear regression. They were used only for determination of SWL.

Table 2 gives the results from tests of various linear models of the relation between SOC and LOI, clay and Fines20. Fines20 was tested in addition to clay because silt‐sized separates may also contain clay minerals. The interaction between LOI and clay for the individual sites was not significant (Highfield, *P* = 0.995; Askov, *P* = 0.193; Lerbjerg, *P* = 0.301). Similarly, the interaction between LOI and Fines20 was not significant (Highfield, *P* = 0.125; Askov, *P* = 0.248; Lerbjerg, *P* = 0.086). Quadratic clay or Fines20 terms were not significant when included in the models for Highfield (clay^2^, *P* = 0.937; Fines20^2^, *P* = 0.581) and Lerbjerg (clay^2^, *P* = 0.439; Fines20^2^, *P* = 0.137). For Askov the quadratic clay term was not significant (clay^2^, *P* = 0.439). However, the quadratic Fines20 term was significant (Fines20^2^, *P* = 0.009), but the assumption of homoscedasticity for the linear regression model was not fulfilled, so the quadratic term was not included in the model. When the intercept of a given model was non‐significant, it was disregarded and the regression forced through the origin. In general, the regression coefficient for LOI was positive, whereas clay and Fines20 had negative regression coefficients when both LOI and clay or Fines20 were included in the models. The best model for each site was taken as the model with the largest *R* ^2^ and smallest AIC. If the intercept was non‐significant, the model without intercept was selected as the best model. Models differing by < 2 in AIC values are not considered significantly different (Burnham & Anderson, 2002). The best models for Highfield included clay, whereas the best models for Askov and Lerbjerg included Fines20. To find the best overall model based on data from all three sites, a model including a linear effect of LOI and clay was tested. However, the plot of residuals for this model showed that it was not fully able to capture the effect of clay across individual sites. Therefore, we tested a model that included a quadratic clay term, and the residual plot revealed a better prediction of clay effect across individual sites. Thus, the best overall model included a quadratic clay expression (model O2.1, Table 3):

(3) $$\mathrm{SOC}=0.513\;\mathrm{LOI}-\left(0.047\;\text{Clay}-0.00025\;{\text{Clay}}^{2}\right).$$

**Table 2:** Parameter estimates, R ^2^ and the Akaike information criterion (AIC) for linear models of the relation between soil organic carbon (SOC) and loss‐on‐ignition (LOI), clay (< 2 μm) and mineral particles < 20 μm (Fines20) for individual sites

Model	Intercept	*P‐*value	LOI / g 100 g^−1^	*P‐*value	Clay / g 100 g^−1^	*P‐*value	Fines20 / g 100 g^−1^	*P‐*value	*R* ^2^	AIC
Highfield										
H1	−1.145	< 0.001	0.526	< 0.001					0.981	−66.6
H2	−0.164	0.322	0.519	< 0.001	−0.037	< 0.001			0.990	−94.9
H2.1	0		0.515	< 0.001	−0.043	< 0.001			0.990	−95.8
H3	0.579	0.083	0.525	< 0.001			−0.034	< 0.001	0.988	−88.2
H3.1	0		0.528	< 0.001			−0.023	< 0.001	0.988	−86.9
Askov										
A1	−0.155	0.244	0.432	< 0.001					0.910	−33.2
A1.1	0		0.379	< 0.001					0.896	−33.5
A2	0.314	0.301	0.461	< 0.001	−0.059	0.107			0.919	−34.8
A2.1	0		0.461	< 0.001	−0.026	0.080			0.925	−35.3
A3	0.440	0.103	0.453	< 0.001			−0.035	0.025	0.939	−38.2
A3.1	0		0.465	< 0.001			−0.013	0.046	0.942	−36.5
Lerbjerg										
L1	−0.103	0.501	0.347	< 0.001					0.959	3.2
L1.1	0		0.335	< 0.001					0.957	1.8
L2	−0.230	<0.001	0.506	< 0.001	−0.026	< 0.001			0.992	−23.0
L3	−0.150	0.028	0.505	< 0.001			−0.022	< 0.001	0.993	−25.0

**Table 3:** Parameter estimates, R ^2^ and the Akaike information criterion (AIC) for linear models of the relation between soil organic carbon (SOC) and loss‐on‐ignition (LOI), clay (< 2 μm) and mineral particles < 20 μm (Fines20). The models are based on data from all three sites

Model	Intercept	*P‐*value	LOI / g 100 g^−1^	*P‐*value	Clay / g 100 g^−1^	*P‐*value	Clay^2^ / g 100 g^−1^	*P‐*value	Fines20 / g 100 g^−1^	*P‐*value	Fines20^2^ / g 100 g^−1^	*P‐*value	*R* ^2^	AIC
O1	−0.280	< 0.001	0.385	< 0.001									0.921	14.9
O2	−0.018	0.720	0.513	< 0.001	−0.046	< 0.001	0.00024	< 0.001					0.987	−123.5
O2.1	0		0.513	< 0.001	−0.047	< 0.001	0.00025	< 0.001					0.988	−125.3
O3	−0.206	0.006	0.507	< 0.001					−0.00956	0.003	−0.00014	< 0.001	0.984	−105.1

**Figure 2:**
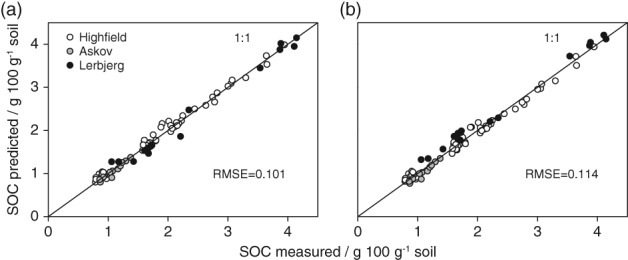
Soil organic carbon (SOC) content predicted by (a) the linear model including loss‐on‐ignition (LOI) and the quadratic clay expression (model O2.1, Table 3 (Equation (3)) and (b) the linear model including LOI and the quadratic mineral particles < 20 μm (Fines20) expression (model O3, Table 3) as a function of the measured SOC content. RMSE, root mean square error.

The inclusion of clay as a predictor increased the variation explained by 7% compared with the simpler model including LOI only (Table 3). The model including Fines20 (O3, Table 3) was almost as good as the model including clay. The interaction term in the models was not significant (O2.1, *P* = 0.172; O3, *P* = 0.991). The clay and Fines20 models predicted SOC with an RMSE of only 0.101 and 0.114 g C 100 g^−1^, respectively, and the predicted versus measured SOC corresponded closely to the 1:1 line (Figure 2).

**Figure 3:**
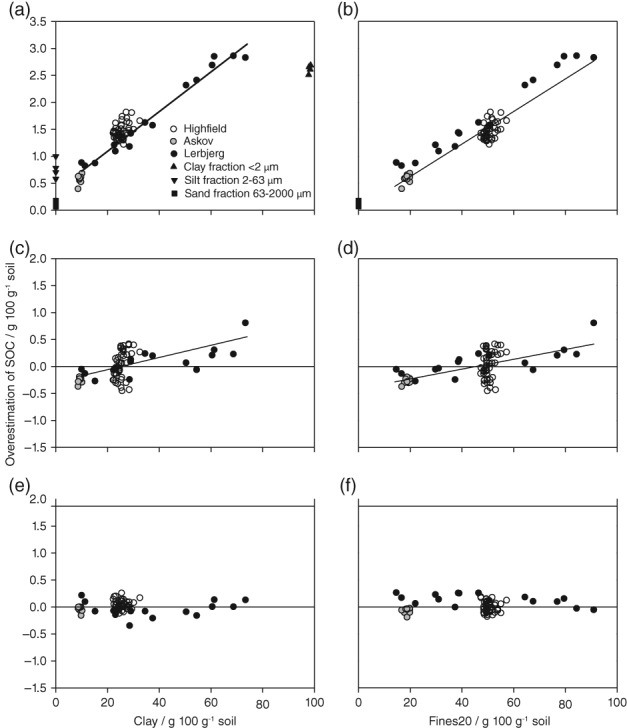
Overestimation (predicted minus measured values) of soil organic carbon (SOC) as a function of clay or mineral particles < 20 μm (Fines20) (a, b) when multiplying loss‐on‐ignition (LOI) with the conventional conversion factor 0.58, (c, d) when estimating SOC by a model including measured LOI (model O1, Table 3) and (e) when estimating SOC by a model including LOI and the quadratic clay expression (model O2.1, Table 3 (Equation (3)) or (f) LOI and the quadratic Fines20 expression (model O3, Table 3). Solid regression lines are indicated if clay or Fines20 had a significant effect on the overestimation of SOC.

The mineral structural water loss (SWL) was estimated by calculating the SOC content as LOI × 0.513 (Equation (3)) and then subtracting the measured SOC content. The SWL from Lerbjerg clay (< 2 μm), silt (2*–*63 μm) and sand (63*–*2000 μm) fractions was 2.11, 0.45 and 0.08 g 100 g^−1^, respectively, with standard deviations of 0.10, 0.18 and 0.04 g 100 g^−1^. The SWL was mainly from the clay fraction, emphasizing the need to include clay or Fines20 in the regression models. When the conventional conversion factor of 0.58 was used, the overestimation of SOC increased significantly with increasing contents of clay (Figure 3a) and Fines20 (Figure 3b).

For soils with large clay and Fines20 contents, the SOC content was overestimated by up to 2.86 g C 100 g^−1^. Predicting SOC from LOI by a regression model overestimated SOC at large clay and Fines20 contents, and underestimated SOC at small contents (Figure 3c,d). Clay and Fines20 had a significant effect on the overestimation of SOC for Highfield (clay, *R* ^2^ = 0.46, *P* < 0.001; Fines20, *R* ^2^ = 0.39, *P* < 0.001) and for all sites (clay, *R* ^2^ = 0.31, *P* < 0.001; Fines20, *R* ^2^ = 0.33, *P* < 0.001). Fines20 had a significant effect on the overestimation of SOC for Askov (*R* ^2^ = 0.42, *P* < 0.022). When the regression model was based on LOI only (O1, Table 3), SOC was underestimated by 0.37 g C 100 g^−1^ and overestimated by 0.81 g C 100 g^−1^ for soils with 9 and 73 g clay 100 g^−1^, respectively. The systematic error disappeared when quadratic clay or Fines20 expressions were included in combination with LOI (Figure 3e,f). The best overall model including LOI and a quadratic clay expression (Equation (3)) predicted SOC with an RMSE of 0.101 g C 100 g^−1^ (Figure 2a).

**Figure 4:**
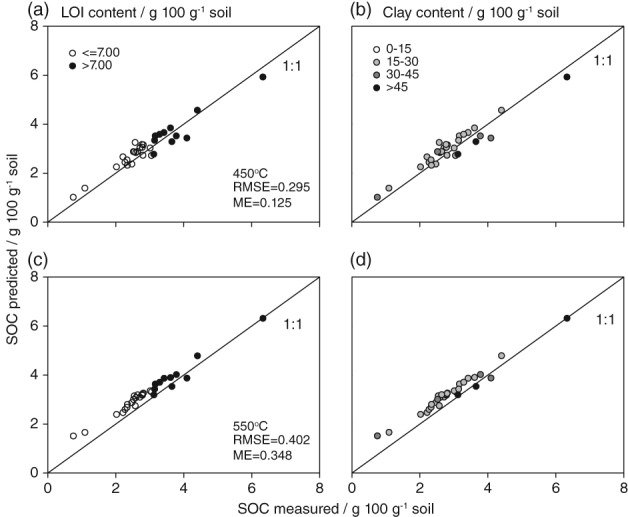
The relation between measured soil organic carbon (SOC) in the evaluation dataset and SOC predicted by a linear model including loss‐on‐ignition (LOI) and the quadratic clay expression (Equation (3)). The SOC predictions were tested with data on LOI based on an ignition temperature of (a, b) 450°C and (c, d) 550°C. Samples are grouped by LOI content (a, c) and clay content (b, d). Based on data published by Grewal et al. (1991). ME, mean error; RMSE, root mean square error.

The data extracted from Grewal *et al*. (1991) for evaluation did not include silt contents and was used only to evaluate the model including the quadratic clay expression (Equation (3)). The range in LOI and SOC contents in the soils evaluated was similar to that of our soils, whereas the range in clay was smaller. Prediction accuracy and bias of Equation (3) were better when the dataset based on LOI‐450 (RMSE = 0.295, ME = 0.125) was used rather than that based on LOI‐550 (RMSE = 0.402, ME = 0.348). Soil organic carbon in the evaluation soils was predicted with an accuracy of ± 0.295 g C 100 g^−1^ at 450°C with Equation (3), and this model had similar predictive capability for small and large contents of LOI and clay (Figure 4).

## Discussion

### Pitfalls

Historical as well as recent estimates of SOC have relied on less accurate analytical approaches such as dichromate oxidation followed by titration and LOI (Bellamy *et al*., 2005; Xie *et al*., 2007; Reynolds *et al*., 2013; Aitkenhead & Coull, 2016). Although these methods involve conversion factors of uncertain scientific foundation (Lettens *et al*., 2007; Pribyl, 2010), they have recently been reported unreservedly as methods for SOC analysis in the Soil Organic Carbon Mapping manual issued by the UN‐FAO (Olmedo *et al*., 2017).

In accordance with Poeplau *et al*. (2015), we found that correcting for RWC is critical to avoid systematic underestimation of SOC. Without correction for RWC, the SOC stock will be underestimated by 2 Mg C ha^−1^ for a topsoil (0–20‐cm depth) with a bulk density of 1.5 g cm^−3^, 2 g C and 30 g clay 100 g^−1^. Converting LOI data by multiplication with the conventional conversion factor 0.58 (Figure 3a,b) overestimates the SOC stock by 45 Mg C ha^−1^ for the same soil. Predicting SOC from LOI with regression model O1 (Table 3) underestimates the SOC stock by 5 Mg C ha^−1^ for a soil with a small clay content (10 g 100 g^−1^) and overestimates the SOC stock by 8 Mg C ha^−1^ for a soil with a large clay content (50 g 100 g^−1^). Predicting SOC content from LOI by a regression model that accounts for clay, increases the accuracy of prediction of SOC stock to ± 3 Mg C ha^−1^ regardless of the clay or Fines20 content. This accuracy could be compared with management‐induced differences in SOC sequestration in an agricultural context, which vary from 0.1 to 1.0 Mg C ha^−1^ year^−1^ (Paustian *et al*., 2016), illustrating that if LOI data are used uncritically the error in the estimate of SOC could easily exceed any management‐induced difference even when adjusted for SWL and RWC. Our study was restricted to arable topsoil from the temperate zone with clay and SOC contents that ranged from 9 to 73 and 0.78 to 4.14 g 100 g^−1^ soil, respectively. Thus, the relations established with the dataset might not be valid for soils under different land use, with different clay mineralogy, subsoils, soils rich in carbonates, and soils with clay and SOC contents outside these ranges (Christensen & Malmros, 1982; Jolivet *et al*., 1998).

### Proposed procedure

Previous studies have shown that if clay content was included in the prediction of SOC by LOI the variance explained was increased (Grewal *et al*., 1991; De Vos *et al*., 2005; Abella & Zimmer, 2007), which corroborates our findings. The difference in the regression coefficients for clay or Fines20 between sites (Table 2) could possibly be a result of differences in clay mineralogy causing differences in structurally bound water. The larger regression coefficient for clay at Highfield than Lerbjerg might relate to a larger kaolinite content in the clay fraction from Highfield. Kaolinite shows a larger loss of water when ignited at 550°C for 4 hours (Sun *et al*., 2009). The presence of negative intercepts for Lerbjerg, when both clay and Fines20 were included (Table 2), might be related to losses other than SOM and mineral structural water loss (e.g. certain salts or free iron) (Pribyl, 2010). The models including clay or Fines20 accounted for structural water loss from clay minerals (Sun *et al*., 2009), which improved the models substantially.

For all sites, the models accounting for clay or Fines20 improved the conversion of LOI to SOC compared with models based on LOI alone. Equation (3) included a quadratic clay expression, which can be interpreted as a decrease in the effect of clay with increasing clay content. Similarly, Spain *et al*. (1982) included a quadratic clay expression in their prediction model. However, further research is needed to explain these observations. The regression model based on the Danish and British soils of the present study (Equation (3)) was able to predict the SOC contents in the New Zealand soils of the evaluation dataset with satisfactory accuracy (LOI‐450, RMSE = 0.295; LOI‐550, RMSE = 0.402). Differences in temperature, sample size, clay mineralogy and SOM characteristics between the evaluation dataset and our dataset might affect model performance.

Where archived soil samples are available, SOC should be determined directly by high‐temperature dry combustion methods, with detection of evolved CO_2_ by infrared or thermal conductivity detectors. However, Arrouays *et al*. (2012) reported that some 40% of the monitoring programmes in the European Union do not archive soil samples. Where LOI has been used to estimate SOC contents and soil samples are no longer available, Equation (3) provides more reliable estimates of SOC stocks for agricultural topsoil provided that LOI data are accompanied by information on soil texture. Equation (3) is valid for the conversion of LOI data that meet the following criteria: ignition temperature of approximately 500°C, ignition duration of 3–6 hours and preferably a sample mass of at least 5 g soil. Additional research that includes a wider range of soil types will increase the applicability of Equation (3).

The regression coefficient for LOI, interpreted as SOM, was similar for all sites when the regression equation accounted for the effects of clay or Fines20 (Table 2). The regression coefficients ranged from 0.45 to 0.52 (=45–52% SOC in SOM), confirming that the conventional conversion factor of 0.58 is too large (Pribyl, 2010). Nevertheless, the so‐called van Bemmelen factor of 1.724 (1/0.58) is still used to convert SOC to SOM (Olmedo *et al*., 2017). The SOC to SOM conversion factor for Highfield, Askov and Lerbjerg was 1.92, 2.02 and 1.94, respectively. Estimating the conversion factor based on all soils gave 1.92. In accord with previous reports (Christensen & Malmros, 1982; Abella & Zimmer, 2007; Chatterjee *et al.,* 2009; Pribyl, 2010), we conclude that the conventional LOI‐to‐SOC conversion factor 0.58 is antiquated and leads to grossly overestimated SOC contents and misleading accounts of SOC stocks.

We acknowledge that other potential sources of error, in addition to the accuracy of the analytical approach, have to be considered when estimating SOC stocks. These potential sources of error include sampling design and intensity, information on the depth of the respective soil layers, and adjustment for stone content and bulk density (Poeplau *et al*., 2017). However, precise estimates of SOC concentrations remain a key issue when establishing credible accounts of SOC stocks (Goidts *et al*., 2009; Schrumpf *et al*., 2011).

## Conclusions

Converting LOI to SOC by the conventional conversion factor 0.58 led to grossly overestimated SOC stocks in agricultural topsoil. When SOC data are based on LOI conversion, we recommend re‐analysis of archived soil samples for SOC by high‐temperature dry combustion methods. Where archived soil samples are not available, accounting for clay content improves the conversion of LOI to SOC considerably.
